# Malaria Prevalence, Spatial Clustering and Risk Factors in a Low Endemic Area of Eastern Rwanda: A Cross Sectional Study

**DOI:** 10.1371/journal.pone.0069443

**Published:** 2013-07-23

**Authors:** Stephen Rulisa, Fredrick Kateera, Jean Pierre Bizimana, Steven Agaba, Javier Dukuzumuremyi, Lisette Baas, Jean de Dieu Harelimana, Petra F. Mens, Kimberly R. Boer, Peter J. de Vries

**Affiliations:** 1 University Teaching Hospital of Kigali, National University of Rwanda, Kigali, Rwanda; 2 Academic Medical Center, Division of Infectious Diseases, Tropical Medicine and AIDS, Amsterdam, The Netherlands; 3 Amsterdam Institute for Global Health and Development, INTERACT Project, Kigali, Rwanda; 4 Royal Tropical Institute/Koninklijk Instituutvoor de Tropen (KIT), KIT Biomedical Research, Amsterdam, The Netherlands; 5 Geography Department, Faculty of Science, National University of Rwanda, Butare, Rwanda; 6 Medical Research Centre, Rwanda Biomedical Centre, Kigali, Rwanda; 7 Department of Internal Medicine, Tergooiziekenhuizen, Hilversum, The Netherlands; Kenya Medical Research Institute - Wellcome Trust Research Programme, Kenya

## Abstract

**Background:**

Rwanda reported significant reductions in malaria burden following scale up of control intervention from 2005 to 2010. This study sought to; measure malaria prevalence, describe spatial malaria clustering and investigate for malaria risk factors among health-centre-presumed malaria cases and their household members in Eastern Rwanda.

**Methods:**

A two-stage health centre and household-based survey was conducted in Ruhuha sector, Eastern Rwanda from April to October 2011. At the health centre, data, including malaria diagnosis and individual level malaria risk factors, was collected. At households of these Index cases, a follow-up survey, including malaria screening for all household members and collecting household level malaria risk factor data, was conducted.

**Results:**

Malaria prevalence among health centre attendees was 22.8%. At the household level, 90 households (out of 520) had at least one malaria-infected member and the overall malaria prevalence for the 2634 household members screened was 5.1%. Among health centre attendees, the age group 5–15 years was significantly associated with an increased malaria risk and a reported ownership of ≥4 bednets was significantly associated with a reduced malaria risk. At the household level, age groups 5–15 and >15 years and being associated with a malaria positive index case were associated with an increased malaria risk, while an observed ownership of ≥4 bednets was associated with a malaria risk-protective effect. Significant spatial malaria clustering among household cases with clusters located close to water- based agro-ecosystems was observed.

**Conclusions:**

Malaria prevalence was significantly higher among health centre attendees and their household members in an area with significant household spatial malaria clustering. Circle surveillance involving passive case finding at health centres and proactive case detection in households can be a powerful tool for identifying household level malaria burden, risk factors and clustering.

## Introduction

From 2005 to 2010, Rwanda achieved the 2005 global community commitment of reducing the malaria burden by at least 50% [1]. During this period, a rapid malaria assessment conducted at 30 out of 40 Hospitals in Rwanda showed reductions of; 74% among confirmed outpatients cases of all ages, 26% in slide positivity rates, 65% among inpatients of all ages, and 55% in malaria deaths [2]. These gains followed rapid scale-up of insecticide-treated mosquito nets (ITNs), indoor residual spraying (IRS), use of artemisinin combination therapies (ACTs) and laboratory confirmation of presumed malaria cases with microscopy (at health facilities) and rapid diagnostic tests (RDTs) (by community health workers) as recommended by WHO’s Roll Back Malaria program [1]. Despite these gains, malaria still causes significant morbidity; 7.8% of all febrile patients presenting at the health centre (HC) had malaria and 12.9% of all age mortality were malaria associated in 2010, with a malaria resurgence recorded in 2009 [2], [3], [4]. These observations highlight the fragility of gains in malaria reduction achieved, especially in areas with a high baseline malaria transmission potential.

Current anecdotal Rwandan national routine data suggests a heterogeneous spatial malaria distribution with the entire population remaining at risk with the exception of the very high altitude zones [3], [5]. Malaria heterogeneity has been reported across the different malaria endemic settings and has been attributed to risk factors including altitude, climate, occupation and socio-economic status [6], [7], [8], [9], [10]. However, at all malaria endemicity levels, and particularly in low incidence areas, malaria tends to cluster in ‘hotspots’ and ‘hot’ populations that become sources of continued infection. We defined a ‘hotspot’ of malaria transmission as ‘a geographical part of a focus of malaria transmission where transmission intensity exceeds the average level’ [11]. In a community, asymptomatic and minimally symptomatic malaria cases, whose symptoms may not be severe enough to seek care, can serve as significant parasite reservoirs for maintaining transmission [7], [8], [12]. Active and timely identification of these hotspots and associated risk factors is essential for targeting interventions to optimize malaria control [13].

Risk factors associated with malaria clustering for which we also investigated include distance of households (HHs) from potential mosquito-breeding sites, house roofing and wall materials and bednet use [7]. In Rwanda, however, there is paucity of systematic HH studies on malaria burden or associated risk factors with most reported data being aggregated routine health facility data. Despite its tendency to underestimate malaria burden, routine data can be helpful in reflecting malaria trends [14], particularly in low malaria incidence settings where the majority of the population access health services from the reporting health facilities. The passively identified health facility cases may reflect area malaria transmission levels in places where malaria cases tend to cluster in time and place. Index cases may also act as entry points to community HHs where identification of hotspots that could be targeted for optimal malaria control. Malaria hotspots may serve to perpetuate residual malaria transmission in low transmission seasons and hinder efforts to eliminate malaria [15].

In this study, we used HC attendees with presumed malaria as entry points for reactive case identification of malaria infections at the HH level. In a two-phase health facility and HH cross-sectional survey, we employed circle surveillance technique to measure malaria burden and evaluate for associated malaria risk factors. We also investigated for spatial malaria clustering using geographical information system (GIS) and spatial statistical techniques [16], [17], [18].

## Materials and Methods

### Ethical Statement

Ethical approval was granted by Rwanda National Ethics Committee. Prior to study initiation, sector and community leaders were informed about the study and their support and verbal consent requested. Written consents were obtained from adult participants and parents/guardians of participating children and from heads of HHs or the oldest person present for the HH surveys.

### Study area

**Figure 1:**
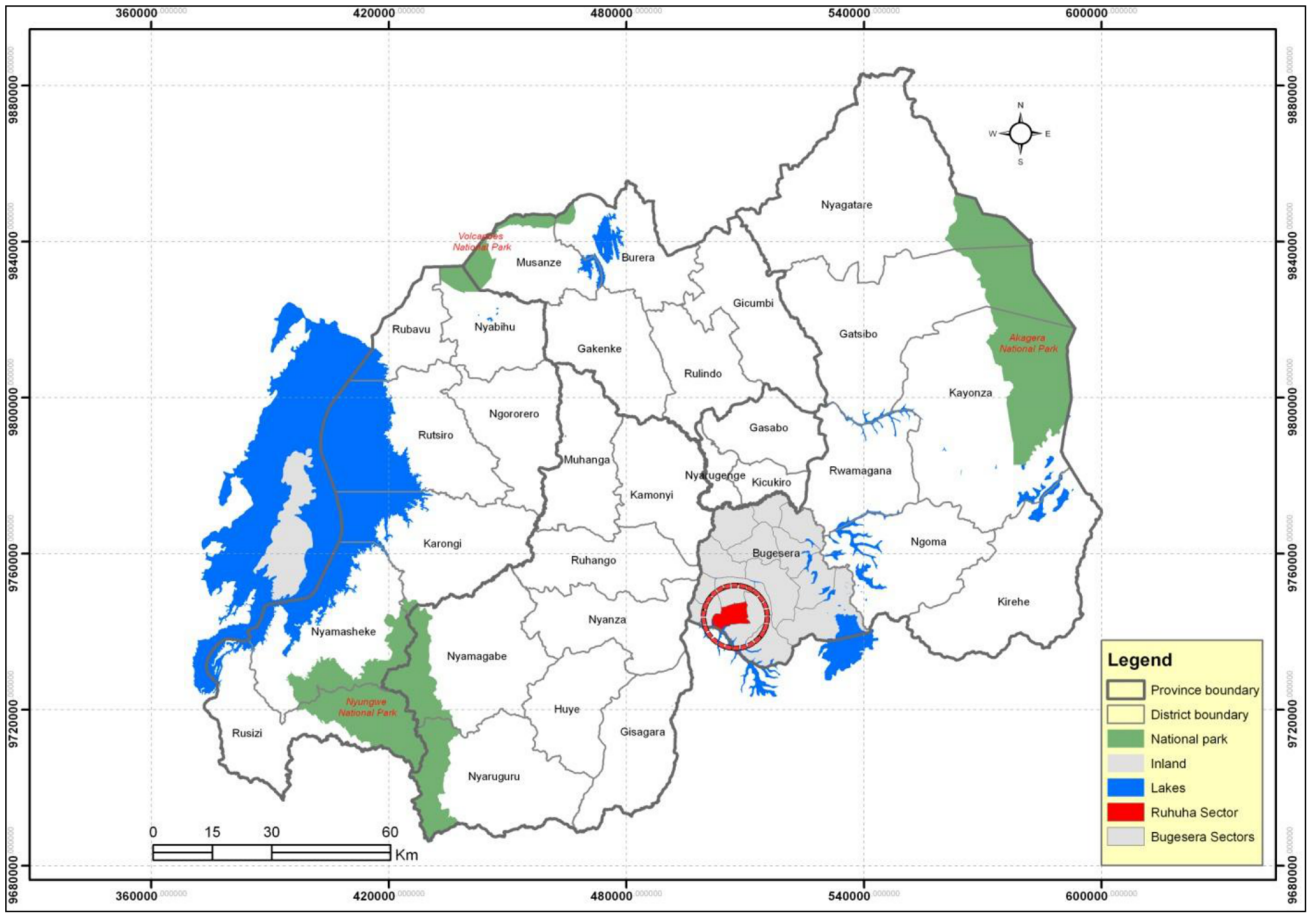
Location of Ruhuha Sector (Red), Bugesera District (Grey) in Rwanda. *Source: MINITRACO/CGIS-NUR, 2001 and NISR 2006.*

The complete survey was conducted in Ruhuha Sector, Bugesera district [19], Eastern Rwanda (Figure1). The sector covers 54 km^2^, has a population of about 19,606 persons living in 4279 HHs. It is predominantly rural and traditionally a high malaria endemic area. Ruhuha sector, surrounded by lowland marshes and water-streams draining into the Akagera River System, is separated from Burundi by Lake Cyohoha in the south.

### Study Design and Participants

A two-phase cross-sectional survey was conducted between April and October 2011. First, a fever survey was conducted among patients presenting at Ruhuha Health Centre (RHC) with a fever or history of fever in the last 24 hours. Patients of all ages were recruited and after signing the informed consent form, malaria diagnosis by microscopy and individual level risk factor data were collected. Thereafter, study participants were invited to participate in a follow-up HH survey where HH level malaria risk factor data was collected and malaria screening for all HH members performed.

### Study Procedures

#### Health centre (HC) fever survey

At the HC, an interviewer-administered questionnaire, adapted from the Measures group Demographic Health Surveys tools and previous studies [20], [21], was administered to adult patients or, in the case of minors, to parents/guardians of the children. The pre-tested questionnaire was administered by study-trained personnel. Data collected included personal demographics, fever characteristics, malaria perception, knowledge and practices including malaria preventive measures, and house structural features (walls and roofs).

#### Preparation of blood films, microscopic examination and quality assurance

To identify malaria among HC attendees, Giemsa stained thick and thin blood films were prepared and read by two independent experienced microscopists at the RHC laboratory. A third microscopist based at National Reference Laboratory (NRL) settled discrepancies between two readings. Parasite negative results were based on screening of 100 microscopic fields at 1000x magnification. Malaria parasites were counted against 200 white blood cells on thick blood films for enumeration of parasite density and thin smears used for species identification. In addition, 10% of all microscopy slides were sent to the NRL for external quality control.

#### Household survey

HC-recruited study participants (regardless of their malaria diagnosis status) who consented to a home visit and provided HH locator information were visited 1 to 4 months later for a follow-up HH survey. At this visit, all HHs were enumerated and assigned a unique identification number. An interviewer-administered questionnaire was used to collect data on HH level malaria risk factor characteristics including, bednet availability, type, integrity and use, HH water sources and environmental factors.

#### Rapid diagnostic test (RDT) screening

In addition to the questionnaire data, all HH members were screened for presence of malaria parasites to measure asymptomatic or minimally symptomatic parasitaemia prevalence using RDTs (*First Response® Combo Malaria Ag (pLDH/HRP2) card test, Premier Medical Corporation Ltd, India*). If HH members were not at home at the time of the survey, they were actively sought out and subsequently screened by the field team. RDTs were performed according to the manufacturer’s instruction by trained field team members. All RDTs used were from one batch that was directly obtained through the manufacturer and stored according to the manufacturer’s recommendations. However, no external quality control was done on these RDTs. Follow-up confirmatory microscopy was provided at the Ruhuha HC for all RDT-positive individuals to confirm accuracy and inform a malaria treatment decision.

#### Mapping households and geographical features

GIS was used to capture, manage and geographically integrate data from different sources. Location data for each HH and key geographical feature was collected using a handheld GPS receiver, GPSMAP 60CSx *(Garmin etrex legend®, Garmin International Inc. USA).* Digitized data from pre-existing shapefiles provided base layers (topography, land use, rivers and surface water) on which study data was overlaid into one geo-database compatible with ArcGIS10. Boundaries shapefiles of administrative units (“cells”), wetlands, water bodies and the elevation contour lines for Ruhuha sector were obtained from the GIS Remote Sensing Training and Research Centre of the National University of Rwanda.

### Statistical analysis

Statistical analysis was performed using STATA software (*version 12, College Station, TX, USA*). Univariate analysis to assess for malaria risk for all variables was done using logistic regression and variables with possible malaria risk (p<0.2) were included in the initial multivariate logistic regression model. HH data was analyzed using generalized estimating equation (GEE) models with adjustment for HH level malaria case clustering. The level of significance for study statistics was p>0.05 and Wald tests were used to quantify variable effects in the model. Possible interaction effects were also assessed for.

#### Spatial clustering

The Kulldorff spatial scan statistic, using SaTScanTM version 9.1.1 software (http://satscan.org), was used to test for spatial clustering of malaria cases and/or to determine whether the cases were distributed randomly over space [Kulldorff & Nagarwalla. [1995]]. HHs, used as the unit of analysis, were located using the Cartesian coordinate system to specify coordinates with the maximum spatial cluster size set at 50% of the population at risk. As in other studies, SatScan generated circular windows of different sizes for detecting clustering [22], [23]. The number of cases in each window was compared to the expected number of cases based on the total number of cases and population size. We used purely spatial analyses based on the Bernoulli probability model that is appropriate for 0/1 event data such as cases/controls. The controls represented the background distribution population. The P-value was obtained from a likelihood ratio test based on Monte Carlo simulation replications of the data set. Spatial scans were performed for both HC attendee and HH member cases. A HC case was defined as being microscopy positive with HC controls defined if they were microscopy negative; a HH member was defined as being a case if they were identified as RDT positive with HH controls defined if they were RDT negative.

## Results

**Figure 2:**
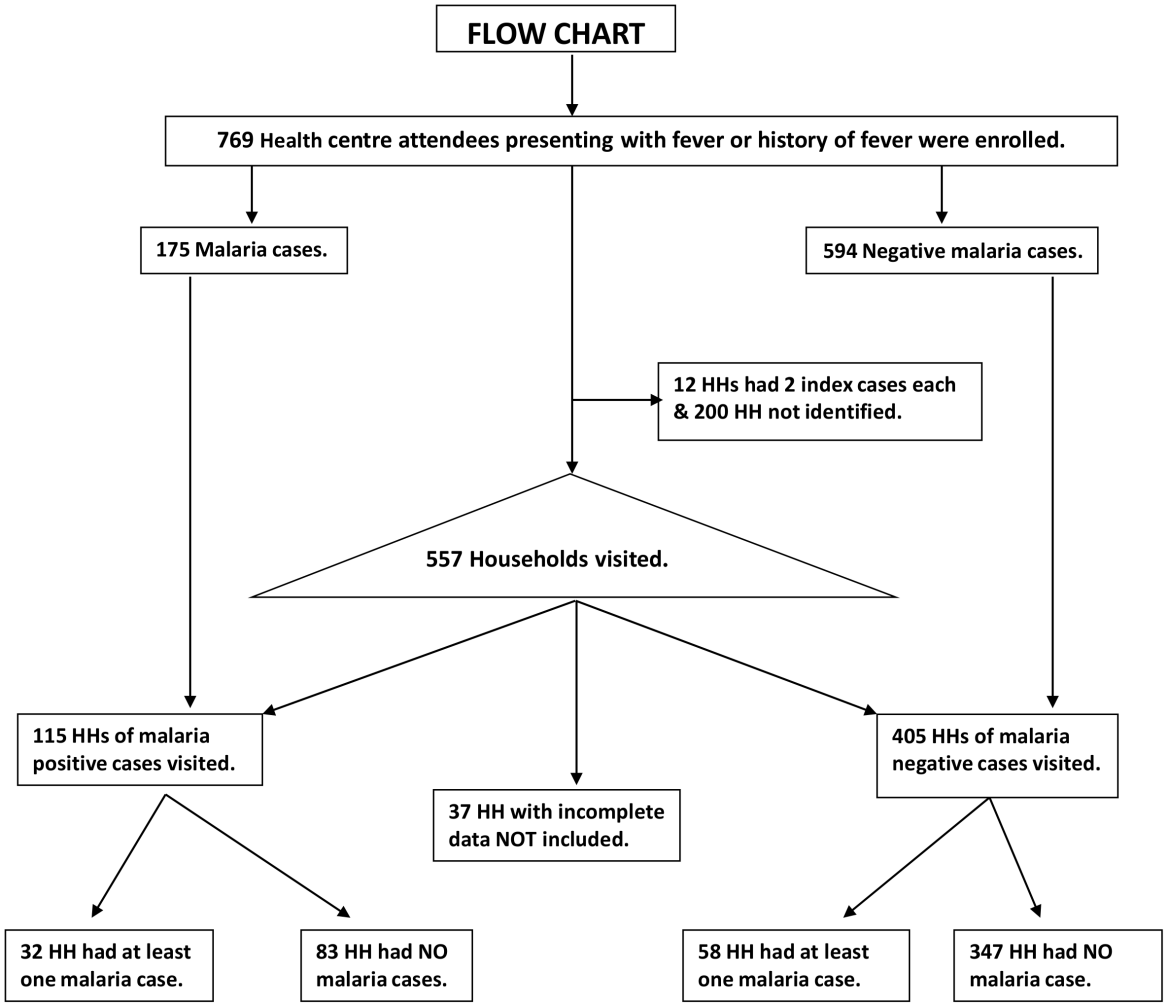
Flow chart of study participant enrolment, malaria screening and participation in a two phase survey.

In total, 769 HC attendees who presented with fever or with a history of fever in last 24 hours at the outpatient clinic were screened. Of the 769; 175 (22.8%) were diagnosed with malaria, 458 (59.6%) were female, 277 (36.0%) were aged <5 years, 147 (19.1%) aged 5–15 years and 345 (44.9) aged >15 years. A flow chart of study participant enrolment, malaria screening and participation is shown in Figure 2. HH visits were planned for all 769 HC attendees. However, because of the long period between HC case enrolment and HH survey (1–4 months versus the planned 2–4 weeks) and the inaccurate location data reported by study participants, the HH survey was not conducted in HHs of 200 index participants. Among HC attendees, malaria prevalence was comparable between those whose HH were not visited (30.5% (CI. 23.4–38.4) and those visited.

Of the 557 (72.4%) surveyed HHs, 520 HHs had complete data. Only data from these 520 HHs were analysed. In total, 2634 HH members were screened for malaria. Of the 2634, 599 (22.2%) were aged <6 years, 763 (28.3%) aged 6 to 15 years and 1331 (49.4%) aged >15 years. Only 90 (17.3%) HHs had at least one member diagnosed with malaria and the overall malaria prevalence (RDT confirmed) was 5.1% (95% CI 4.34–6.03). All visited HHs had ≥1 bednet and in total, 873 bednets were observed. HH bednet and indoor residual spraying coverage by self-report were 97.1% and 98.2%, respectively. Basic knowledge about malaria was high, with 696 (91%) reporting bednets as the principle malaria preventive measure while 748 (97.3%) reported that fever was the principal malaria symptom. Interestingly, 447 (82.5%) of HHs visited had bednets in their possession but these were not physically hung (Table 1).

**Table 1:** Reported and observed bednet characteristics.

Characteristics of bednets as reported by HC attendees.						Characteristics of bednets observed during HH visits (n = 557 HHs)			
*Reported malaria preventive measures* *used in HH N (%)*		*Are bednets in your HH treated? N (%)*		*How many holes are in your bednets? N (%)*		*Observed N (%) of hanged bednets*		*Observed Bednet in HH N (%)*	
Bednets	696 (82.6)	No	21 (2.92)	No holes	649 (91.28)	0	447 (82.47)	0	88 (11.4)
Clear bushes	66 (7.8)	Yes	615 (85.65)	1-10 holes	31 (4.36)	1	93 (17.16)	1	279 (36.3)
No protection	64 (7.6)	Do not know	55 (7.66)	> 10 holes	4 (0.56)	> 1	2 ( 0.37)	2	240 (31.2)
Others	11 (1.3)	Missing	27 (3.76)	Missing	27 (3.8)			≥ 3	134 (17.4)
Missing	6 (0.7)							Missing	28 (3.7)

### Univariate Analysis

Results of univariate analysis for individual and HH (after adjusting for possible house-level clustering of cases) risk factors are displayed in Tables 2 and 3, respectively. Malaria risk among HC attendees was associated with both age and reported bednet ownership. Compared to children ≤5 years, malaria prevalence was three times higher in the 6–15 year olds while a reported ownership of ≥4 bednets was associated with a significant protective effect. HC attendees were evaluated for symptoms predictive of having clinical malaria. Having a measured fever (≥37.5°C) at presentation was associated with higher odds of malaria risk than no fever. Similar to HC cases, malaria risk among their HH members was significantly associated with age and observed bednet coverage. Additionally, HH members living in houses made of wood/mud/tent, when compared to those HH members living in dwellings whose walls were made of stone or bricks, and HH ownership of an in-house open water vessel were associated with higher odds of malaria.

**Table 2:** Health facility attendee characteristics and malaria risk factors.

Baseline characteristics	N (%)	HC attendees with malaria (n = 175) (%)	HC attendeeswith No malaria(n = 584) (%)	Univariate Analysis	Multivariate Analysis
***Age group***					
≤5 years	277 (36.02)	57 (32.6)	220 (37.0)	1.0	1.0
6−15 year	147 (19.12)	57 (32.6)	80 (13.5)	2.444 (1.572–3.801), <0.0001	3.02 (1.890–4.824), <0.0001
≥16 years	345 (44.86)	61 (34.8)	284(49.5)	0.829 (0.555–1.239), 0.36	1.027 (0.663–1.591), 0.906
***Gender***					
Male	311 (40.44)	73 (41.7)	238(40.1)	0.934 (0.663–1.316), 0.696	
Female	458 (59.56)	102 (58.3)	356(59.9)	1.0	–––-
***Measured temperature at HC***					
<37.5	381(49.5)	69(39.4)	312(52.5)	1.0	1.0
≥37.5	388 (50.5)	106(60.6)	282(47.5)	1.700 (1.206–2.396), 0.002	1.636 (1.119–2.392), 0.011
***When did fever episode start***					
Today	47 (6.11)	10(5.7)	37(6.2)	1.0	–––-
Yesterday	494 (64.24)	109(62.3)	385(64.8)	1.048 (0.505–2.174), 0.901	
Day before yesterday	162 (21.07)	46(26.3)	116(19.5)	1.467 (0.674–3.193), 0.334	
Long ago	66 (8.58)	10(5.7)	56(9.5)	0.661 (0.250–1.743), 0.402	
***Malaria episode in past 12 months***					
None	520 (67.62)	105	415	1.0	1.0
1–3 episodes	232 (30.17)	64	168	1.506 (1.052–2.156), 0.025	1.408 (0.959–2.068), 0.081
>3 episodes	17 (2.21)	6	11	2.156 (0.780–5.964), 0.139	2.163 (0.737–6.346), 0.160
***Does HH own at least 1 bednet***					
Yes	742 (96.61)	167	575	0.653 (0.279–1.530), 0.327	
No	26 (3.39)	8	18	1.0	–––-
***Reported number of bednets in HH***					
One	88 (11.88)	24	64	1.0	1.0
Two	279 (37.65)	65	214	0.810 (0.470–1.397), 0.449	0.654 (0.372–1.150), 0.141
Three	240 (32.39)	59	181	0.869 (0.500–1.512), 0.620	0.687 (0.386–1.220), 0.200
≥ Four	134 (18.08)	19	115	0.441 (0.224–0.865), 0.017	0.352 (0.175–0.707), 0.003

**Table 3:** Household characteristics and malaria Risk factors.

Variable	Frequency (%)	HH with ≥ one malaria case	HH with No malaria case	Univariate Analysis	Multivariate Analysis
***Gender***					
Female	1167 (44.3)	64	1,091	1.247 (0.894− 1.740), 0.194	1.191 (0.852−1.667),0.306
Male	1467 (55.7)	66	1,408	1	1.0
***Age group***					
≤5 years	589 (22.40)	26	563	1	1.0
6−15 years	742 (28.22)	71	671	2.398 (1.528−3.766), <0.0001	2.437 (1.543−3.847), <0.0001
≥16 years	1,298 (49.37)	33	1,265	0.586 (0.350−0.982), 0.042	0.584 (0.344−0.992), 0.047
***HH member association with***					
Negative index case	2,047 (77.86)	77	1,970	1	1.0
Positive Index case	582 (22.14)	53	529	2.557(1.608−4.066),<0.0001	1.267 (1.068−1.503), 0.007
***Observed No of bednets***					
One bednet	256 (9.77)	22	234	1	1.0
Two Bednets	2,212 (84.46)	99	2,113	0.490 (0.204−1.179), 0.111	0.456 (0.202−1.029), 0.059
Three Bednets	55 (2.10)	2	53	0.526 (0.217−1.274), 0.155	0.461 (0.207−1.024), 0.057
≥ Four bednets	96 (3.67)	7	89	0.367 (0.143−0.940), 0.037	0.384 (0.165−0.892), 0.026
***House wall material***					
Bricks and stones	364 (70)	58	306	1	1.0
Wood/mud/tent	156 (30)	32	124	1.324 (1.134−1.546), <0.0001	1.288 (1.082−1.534), 0.004
***Type of HH roof material***					
Corrugated Iron sheets	457 (87.9)	79	378	1	1.0
Grass thatched/tent/others	63 (12.1)	11	52	0.849 (0.662−1.088), 0.196	0.837 (0.636−1.102), 0.204
***Was IRS of HH walls done***					
Yes	490 (94.4)	88	402	1	–––-
No	30 (5.6)	3	27	1.033 (0.742−1.435), 0.852	
***Presence of outside water source***					
Yes	205 (36.80)	45	150	1	–––-
No	352 (63.20)	45	280	0.762(0.489−1.190), 0.232	
***Have an open water vessel in HH***					
Yes	171 (32.88)	35	136	1	1.0
No	349 (67.12)	55	294	0.666(0.425−1.045), 0.077	0.712 (0.351−1.444), 0.347
***Green environment around HH***					
Very green (grass & trees)	300 (53.86)	47	244	1	1.0
Moderate green (only grass)	174 (31.24)	35	124	1.720 (1.076−2.750), 0.023	1.412 (0.691−2.886), 0.344
No grass at all	83 (14.90)	8	62	0.825(0.383−1.773), 0.622	0.578 (0.218−1.528), 0.269
***Does your HH have Electricity?***					
Yes	34 (6.10)	4	25	0.512(0.154−1.710), 0.277	0.634 (0.248−1.617), 0.340
No	523 (93.90)	86	405	1	1.0
***Have Domestic Animals in HH?***					
Yes	376(67.50)	62	291	0.931 (0.564−1.537), 0.779	
No	181 (32.50)	28	139	1	–––-

### Multivariate Analysis

At the individual level, an adjusted multivariate logistic regression model showed significantly higher odds of clinical malaria risk among children aged 5–15 years (OR = 3.02, P value <0.0001) but a protective effective was noted in those with a reported ownership of 4 of more bednets (OR = 0.352, P value 0.003). Having a fever (≥37.5°C) was predictive of having clinical malaria (OR = 1.64, P value 0.011). House level malaria risk remained significantly associated with age, type of material HH dwelling was made of, observed bednet coverage and malaria status of index case after adjusting for malaria case clustering in HHs (Table 3). Compared to the ≤5 year age group, malaria risk was significantly higher among the 6–15 year age group (OR = 2.44, P-value <0.0001) but interestingly lower, albeit with a borderline statistical significance, among the ≥16 year age group (OR = 0.58, P-value 0.047). Living in dwellings made of wood or mud or tent material was associated with a higher malaria risk while an observed ownership of ≥4 more bednets was associated with a protective effect.

### Malaria Clustering

**Figure 3:**
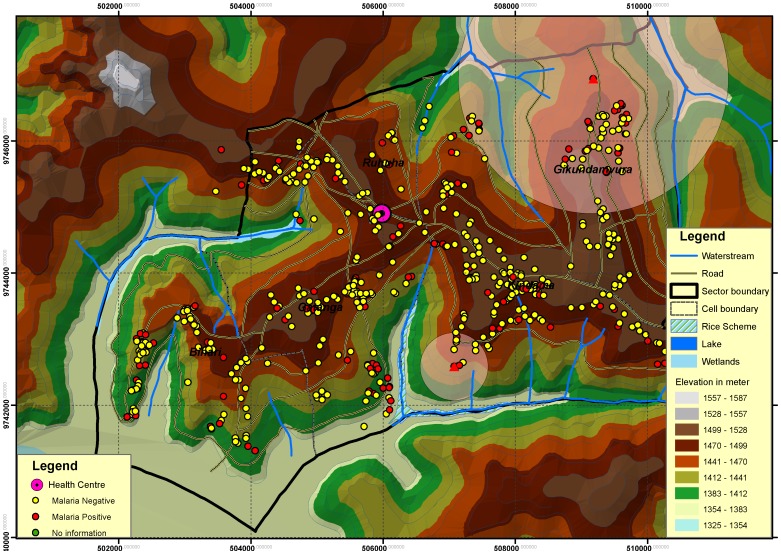
^¥^ Spatial malaria clusters and location of Hhs. (Yellow dots - control HH with no malaria infected case and small Red dots - case HH with at least one malaria infected case) in Ruhuha sector. *^¥^The used administrative boundaries and geographic features shape files were obtained from the Centre for Research and Training in GIS and Remote Sensing of the National University of Rwanda.*

Malaria positivity among HC attendees was significantly correlated with a HH having at least one confirmed member (OR = 2.31, *P = *0.001) but no spatial clustering for HC malaria cases was observed. However, three clusters of HHs with significantly higher risk than expected RDT tested members were identified (Table 4). These HH clusters were located; 1. North East (radius of 2.04 Kilometers (Kms), relative risk of 3.40 and P value 0.0001), 2. South (radius of 0.51 Kms, relative risk of 5.6, (P value 0.0001), and 3. A smaller cluster (not indicated in Figure 3) of only one HH (where 4 of its members tested RDT positive) with a relative risks of 20.8, P value 0.002 (Figure 3). Two of these clusters (1 and 2) were located next to water-based agro-ecosystems.

**Table 4:** Spatial clustering of the more than expected household cases.

Cluster	Year	No of HHs in cluster	P - value	Observed No ofHH cases	ExpectedNo of Cases	Relative risk
1	2011	60	0.00002	38	14.064	3.413
2	2011	11	0.00015	16	3.169	5.622
3	2011	1	0.00247	4	0.198	20.808

## Discussion

In this study, members of HHs where the index case had clinical malaria showed 1.3 times greater odds of being malaria infected compared to members of HH where the index patient was malaria negative. Comparable findings of a greater risk for malaria infection among HH members of a HC identified clinical malaria case have been shown by Stresman et al. (2010) in Zambia [24]. These findings support the value of circle surveillance as a useful tool for studying HH level malaria burden, risk factors and clustering. In this study, slide/RDT positivity rates of 22.8% and 5.1% among HC malaria presumed cases and HH based asymptomatic cases respectively were found. This demonstrates that circle surveillance can show differences in HH malaria risk and clustering, even in areas of high malaria prevalence as in Ruhuha. A part from living in a HH where the index case had malaria, risk factor analysis identified participant’s age and a reported ownership of a ≥4 bednet as variables that, either alone or in unison, significantly influenced malaria risk.

Compared to children aged <5 year, older children and adults had a higher risk of parasite carriage, for both HC attendees and HH members groups. This is in contrast to previous findings of a higher malaria risk in children <5 years [25]. However, a shift to higher malaria risk among older age groups has been reported after the increased coverage with insecticide-treated bednets and the observed follow-up reduced malaria transmission in some communities [26], [27]. The reductions in malaria transmission may decrease the risk of malaria inoculation and infections leading to an increase in the age at which malaria infections are first acquired. Additionally, there is a greater likelihood of younger age groups (<5 year olds) using malaria preventive bednets compared to their older siblings, although this data was not collected in this study [26].

In this study, the reported and observed ownership of bednets was associated with significant malaria protective effect. This protective effect of insecticide-treated mosquito net use has also been affirmed in multiple previous studies [28]. Ruhuha sector is a traditionally high transmission setting with high bednet coverage. This high coverage follows the government’s massive free bednet distribution after campaigns run between 2009 to 2011 in which government aimed to achieve universal bednet coverage [5], [29], [30]. Study Participants reported a good level of knowledge of malaria symptoms, transmission and preventive measures with over 82% of respondents reporting use of bednets the night before the survey. However, in only 18% of visited HHs was a bednet found physically hung onto a bed or a sleep space suggesting that bednet use may be sub-optimal. Possible reasons for sub-optimal bednet use may be associated with local house structures and/or sleeping arrangements for the HH members. Most houses in Ruhuha have 1–2 bedrooms with limited structures on which to hang bednets. Additionally, most occupants share sleeping spaces on the floor. These factors may complicate use of available bednets and partially explain the low bednet hanging rates observed and limited bednet protective effects in HHs with bednets. Studies exploring how to optimise bednet usage and effectiveness are recommended.

In this study and others, the quality of housing, apart from being an indicator of HH economic status has been reported, to influence the ease with which mosquitoes can enter and hide in a home and hence contribute to malaria risk [7], [31], [32]. Occupants of houses with walls made of mud/grass/wood had 1.3 times greater odds (P value 0.016) of having at least one malaria case more than those living in houses with walls made of brick or stone. However, interventions to address type of housing as a malaria risk factor are complex and difficult to achieve and are rarely components of public health programs. A current campaign in Rwanda to phase out grass-thatched houses (locally known as *“nyakatsi”)* and replace them by houses made of brick and iron sheet roofscould impact malaria transmission.

For high transmission countries where essential clinical services are adequately available, the transition from control to elimination is recommended at SPR of <5% [12]. Achieving pre-elimination levels in Ruhuha, given current SPR of >22%, will probably require introduction of novel area-relevant interventions to supplement existing control tools (mainly ITNs and IRS). As malaria transmission declines, a community-based evaluation of transmission intensity and size of infectious reservoir will be required. In this study, malaria prevalence among HH members by RDT was 5.1%. However, since RDTs have a lower sensitivity, as compared for example to molecular tools, the level of true malaria infection prevalence among the predominantly asymptomatic carrier HH members, may have been underestimated [33], [34]. In addition, the HH survey was conducted 1 to 4 months, rather than the planned 2–4 weeks, after the initial HC-based fever survey. This delay may have complicated a fair comparison of malaria risk between HC index cases and their HH members. Ruhuha sector is served by only one HC managed primarily by community health workers with most children ≤5 years. The area population is therefore challenged by inadequate access to health care. Consequently, malaria data reported from this health centre may underestimate the population malaria burden. This further complicates a fair comparison of health centre versus HH level malaria risk [13].

Two hundred HHs could not be identified due to; wrong directions, non-existing HHs and, possibly, out of area study participants who gave wrong data. Given the delay in the follow-up HH surveys and the significant loss to follow-up of index cases, a repeat robust reactive case identification study to assess for clustering, particularly in areas of lower malaria transmission intensity, is recommended [12], [15]. In this study, HH cases were RDT confirmed while HC cases were microscopically confirmed in keeping with national malaria guidelines. However, no quality control for used RDTs was conducted. Also, being a cross- sectional survey, malaria burden reported could not reflect seasonal malaria trends and prospective malaria incidence risk.

Apart from the study limitations reported above, this study showed that having malaria among HC attendees was significantly predictive of finding at least one malaria-infected case among his/her HH members (OR = 2.4, P value - 0.001) suggesting that HC-based passive case identification can be a feasible entry point for identifying community hotspots of malaria infection. Guidelines on how to manage asymptomatic and minimally symptomatic RDT positive cases identified through active case detection are lacking and would be required in the event that circle surveillance is implemented in the future. The currently recommended first line treatment for uncomplicated malaria in Rwanda is Artemether – Lumefantrine (AL). AL has anti-gametocidal effects and an ability to reduce asexual parasitaemia levels and infectivity among malaria-infected individuals [35], [36], [37]. It is plausible that AL can be used among asymptomatic and minimally symptomatic cases to clear local reservoir pools and reduce their malaria transmission potential.

Significant spatial clustering for HH cases (but not HC cases) with the clusters located near water-based agro-ecosystems is an interesting finding. The bigger cluster (radius of 5 km) is neighboring marsh lands where traditional rice cultivation is done (North East), while the smaller cluster (0.5 km radius) is located between multiple water streams and Lake Cyohoha in the south where vegetable and other agriculture crops are grown. We speculate that these water agro-ecosystems may provide significant reservoirs for mosquito breeding and hence increased vector intensity for malaria transmission. This finding suggests that future malaria control efforts should consider targeting potential breeding sites and engaging farming communities. To this end, an entomological evaluation of mosquito breeding capacity and endemicity may guide introduction of integrated vector management practices while community-based environmental management approaches for malaria control, as shown to be effective in settings comparable to Ruhuha, may be two potential effective area relevant strategies to employ [32]. To achieve malaria pre-elimination status in Ruhuha, the bednet and IRS strategies, which are principally used, may need to be complimented by interventions that target area breeding sites and malaria risk factors identified through spatial clustering technique as was done in this study may be required [12].

### Conclusion

In this study, HC malaria confirmed cases were significantly associated with finding at least one malaria-infected case among their HH members. Reactive case finding, by linking HC-identified passive cases to actively identified HH malaria infection, is a potentially powerful surveillance system for identifying HHs with significant malaria risk and detecting asymptomatic carriers. Especially in low transmission settings, identifying and treating asymptomatic carriers is key in interrupting transmission. Therefore, circle surveillance, when combined with knowledge on the individual, the HH and the environmental malaria risk factors in a given community, can aid detection of hotspots and inform use of targeted malaria control strategies.
